# Age as A Predictor of Embryo Quality Regardless of The Quantitative Ovarian Response

**DOI:** 10.22074/ijfs.2016.4579

**Published:** 2016-11-11

**Authors:** Juliano Brum. Scheffer, Bruno Brum. Scheffer, Rafaela Friche de Carvalho, Joyce Rodrigues, Michael Grynberg, Daniel H. Mendez Lozano

**Affiliations:** 1 Brazilian Institute of Assisted Reproduction, Belo Horizonte, Brazil; 2 Department of Reproductive Medicine, Hospital Jean Verdier (AP-HP), University Paris XIII, and INSERM, Paris, France; 3 School of Medicine, Tecnológico de Monterrey and Center for Reproductive Medicine CREASIS San Pedro Monterrey, México

**Keywords:** Age, Anti-Mullerian Hormone, Follicle-Stimulating Hormone

## Abstract

**Background:**

One determining factor of a successful *in vitro* fertilization (IVF) cycle is embryo quality. The aim of the present study was to evaluate associations of embryo quality and reserve markers like age, FSH and AMH.

**Materials and Methods:**

In this prospective study, 120 infertile women, aged 21-44 years, undergoing routine exploration during an unstimulated cycle preceding assisted reproductive technology (ART) at our center were studied prospectively, from February 2011 to December 2014. Descriptive parameters and patient characteristics were reported as mean (SD) or median (range) depending on the distribution. Student’s t test was performed for continuous variables, Wilcoxon and Pearson’s Test were used for not distributed variables and Fisher’s Test was performed for categorical variables. P<0.05 was considered statistically significant.

**Results:**

Overall, at the time of investigation, patients had a mean age of 33.03 ± 4.15 years old. On cycle day three, serum anti-Mullerian hormone (AMH) level was 3.50 ± 1.54 ng/mL, serum follicle-stimulating hormone (FSH) level was 6.29 ± 1.53 mUI/ mL, at baseline, women had 16.57 ± 7.0 antral follicles. The mean of collected oocytes was 11.80 ± 5.25, embryo I+II was 2.46 ± 2.11. A greater number of embryos I+II was observed in young patients. By evaluating 120 patients, a significant relationship was observed between age and FSH (r=0.24, P=0.01), age with AMH (r=-0.22, P=0.02), age with collected oocytes (r=-0.23, P=0.03) and age with embryo I+II (r=-0.22, P=0.03). A significant relationship was also observed between antral follicle count (AFC) and AMH (r=0.29, P=0.01), AFC and the number of transferred embryo (r=-0.18, P=0.03), AFC and total dose of the drugs (r=-0.23, P=0.03). Significant relationship of FSH with total dose of drugs (r=0.19, P=0.02) was also observed. In addition, we determined significant relationships between AMH and the number of collected oocytes (r=0.38, P=0.01), AMH and the number of metaphase II oocytes (r= 0.35, P=0.01), AMH and the number of embryo (r=0.19, P=0.04) as well as AMH and total dose of the drugs (r=-0.25, P=0.01).

**Conclusion:**

Commonly used clinical markers of ovarian reserve are reflection of the ovarian reserve, while the outcome measurements of ART and age are the best predictors of embryo quality.

## Introduction

A classic report on the effect of female age on fertility found that the percentage of women, who using no contraception remained childless, were increased steadily according to their age of marriage: 6% at the age of 20-24 years, 9% at the age of 25-29 years, 15% at the age of 30-34 years, 30% at the age of 35-39 years, and 64% at the age of 40-44 years (1).

According to the 1999 Assisted Reproductive Technology Success Rates (ARTSR), the percentage of clinical pregnancies (gestational sac as imaged with sonography) which is failed to result in a live birth was 14% for women with younger than 35 years of age, 19% for those with 35-37 years of age, 25% for those with 38-40 years of age, and 40% in those with older than age of 40 years (2). 

The age-associated decline in female fecundity as well as increased risk for spontaneous abortion, are largely attributable to abnormalities in the oocyte. The meiotic spindle in the oocytes of older women frequently exhibits abnormalities in chromosome alignment and microtubular matrix composition (3). Higher rates of single chromatid abnormalities in oocytes (4), as well as aneuploidy in preimplantation embryos (5) and ongoing pregnancies, are observed in older women. The higher rate of aneuploidy is a major cause of increased spontaneous abortion and decreased live birth rates in women of advanced reproduc- tive age. 

Evaluation of ovarian reserve has been the focus of a substantial amount of clinical research during the past several years (6-10). A number of tests have been proposed and evaluated that may be used to prognosticate ovarian responsiveness to exogenous gonadotropin stimulation, quality of the oocytes, subsequent implantation and pregnancy rates (PR) (6-9). The prognostic value of these tests has been clearly demonstrated by a number of investigators in a wide variety of settings (9, 10). The main markers of ovarian reserve are age, basal follicle-stimulating hormone (FSH), anti-Mullerian hormone (AMH) and/or basal antral follicle count (AFC) that are valuable for determining stimulation protocols and predicting assisted reproductive technology (ART) outcome (11-22). 

One decisive factor of a successful *in vitro* fertilization (IVF) cycle is embryo quality. Currently, embryo quality is determined by direct visualization of an embryo by an embryologist, who assesses the morphological appearance or markers, to evaluate embryo health and quality (23). 

The aim of the present prospective study was to evaluate the associations of embryo quality and ovarian reserve markers like age, FSH, AMH after stimulation with gonadotropin-releasing hormone agonist- for the respective treatment. 

## Materials and Methods

### Subjects

120 infertile women (aged 21-44 years) undergoing routine exploration during an unstimulated cycle and preceding ART were studied at IBRRA, Brazil prospectively from February 2011 to December 2014. All patients met the following inclusion criteria: i. Both ovaries present, ii. No current or past diseases affecting ovaries, gonadotropin or sex steroid secretion, clearance or excretion, iii. No current hormone therapy, iv. Adequate visualization of ovaries at transvaginal ultrasound scans, and v. Total number of small antral follicles (3-12 mm in diameter) between 1 and 32 follicles, including both ovaries. All patients signed an informed consent form for this analysis. 

### Protocol

The patients received leuprolide acetate (Lupron, Abbott, France), and the gonadotropin-releasing hormone (GnRH)-agonist was initiated at a dose of 2.0 mg/day during the midluteal phase with approximately a 5-days overlap with the OCP (Diane 35, Schering, Brasil). Pituitary down-regulation was monitored and patients with adequate pituitary desensitization started their recombinant FSH regime (Gonal-F, Merck- Serono Pharmaceuticals, Italy) and dose of the GnRH-agonist was reduced to 1.0 mg/day. FSH was started with dosages between 150 and 300 IU daily for 4 days, with or without human menopausal gonadotropin (hMG, Menopur, Ferring Pharmaceuticals, Germany). Thereafter, dose of the FSH was individually adjusted according to the estradiol (E_2_) response and vaginal ultrasound findings. 

When two follicles reached to ≥16-18 mm, 250 mg, recombinant human chorionic gonadotropin (Ovidrel, Merck-Serono Pharmaceuticals, Italy) was administered and oocyte retrieval occurred 35-36 hours later. 

Intracytoplasmic sperm injection (ICSI) was routinely performed in all of the fertilization procedures. Fertilization was evident when two pronuclei were observed. Embryos were cultured until the day of transfer (day 3) in IVF Global® media (Life Global, Canada) supplemented with 10 % synthetic serum substitute (SSS) and graded by Veeck’s criteria (24) before transfer.

Veeck’s morphological grading system was modified and adopted for day 3 embryo scoring, as follows: grade I=8 cells, blastomeres of equal size and no cytoplasmic fragments; grade II=8 cells, blastomeres of equal size and <20% cytoplasmic fragments; grade III=8 cells, uneven blastomere sizes and no cytoplasmic fragments; and grade IV=4 or 8 cells with >20% fragmentation. The same embryologist performed all embryology and embryo scoring, in this study.

Embryo transfer (ET) number was determined using the Federal Council of Medicine-Brazil (FCM) guidelines. Embryo grade I, II and III was transferred. Luteal phase was supported with micronized P4, 600 mg/day, administered continuously by vaginal route, starting on the evening of ET. 

### Hormonal measurements and ultrasound scans 

On the third day of cycle preceding COH, each woman underwent blood sampling by venipuncture for serum AMH, and FSH measurement and a transvaginal ovarian ultrasound scan was performed for follicle measurement. 

Serum levels of AMH and FSH were deter- mined using an automated multianalysis system with chemiluminescence detection (ACS-180, Bayer Diagnostics, Puteaux, France). Serum AMH levels were determined using a second generation enzyme-linked immunosorbent assay. Intra- and inter-assay coefficients of variation were <6% and <10%, respectively, with the lower detection limit at 0.13 ng/mL and linearity up to 21 ng/mL for AMH. For FSH, functional sensitivity was 0.1 mIU/mL, and intra-assay and inter-assay CV were 3 and 5%, respectively. Ultrasound scans were performed using a 3.7-9.3 MHz multifrequency transvaginal probe (RIC5- 9H, General Electric Medical Systems, France) by a single operator who was blinded to the results of hormone assays. 

The objective of ultrasound examination was to evaluate the number and size of small antral follicles. Follicles measuring of 3-12 mm in mean diameter (mean of two orthogonal diameters) in both ovaries was considered. 

To optimize the reliability of ovarian follicular assessment, the ultrasound scanner was equipped with a tissue harmonic imaging system, which allowed improved image resolution and adequate recognition of follicular borders. Intra-analysis CV for follicular and ovarian measurements was <5%, and their lower limit of detection was 0.1 mm. In an effort to evaluate the bulk of granulosa cells in both ovaries, we calculated the mean follicle diameter (cumulative follicle diameter divided by the number of follicles measured 3-12 mm in diameter in both ovaries) and the largest follicle diameter. 

### Ethical approval 

Written informed consent was obtained from all participants before inclusion. The study was approved by Brazilian Institute of Assisted Reproduction Ethical Committee, Brazil. 

### Statistical analysis 

Descriptive parameters and patient characteristics were reported as mean (SD) or median (range) depending on the distribution. Student’s t test was performed for continuous variables, Wilcoxon and Pearson’s Test were used for not distributed variables and Fisher’s Test was performed for categorical variables. P<0.05 was considered statistically significant. 

## Results

**Fig.1:**
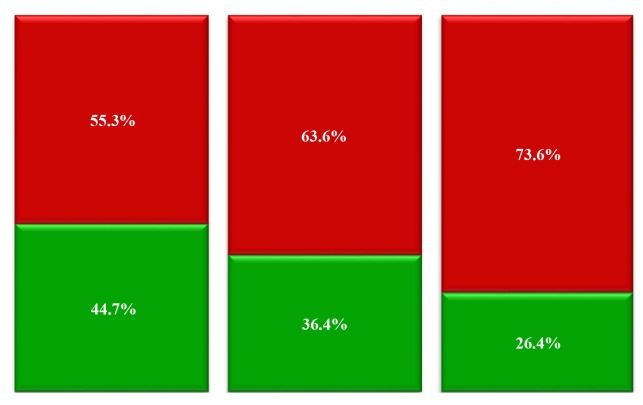
Comparison of different groups. Fisher’s Test (P=0.027). Green: embryos I+II and Red: embryos III+IV

Overall, at the time of the investigation, patients had a mean age of 33.03 ± 4.15 years old, body mass index (BMI) 22.78 ± 4.01 kg/m^2^ , and infertility length of 3.1 ± 2.36 years. 67% of individuals had regular cycles. On cycle day 3, serum AMH level was 3.50 ± 1.54 ng/mL, serum FSH level was 6.29 ± 1.53 mUI/ mL, at baseline, women had 16.57 ± 7.0 antral follicles. The mean day of stimulation was 12 ± 1:41 days and the mean total dose of drugs was 3382.29 ± 778.06 IU. The mean collected oocytes was 11.80 ± 5.25, metaphase II oocytes was 10.64 ± 5:07, embryo grade I was 0.32 ± 0.63, grade II was 2.14 ± 1.90 embryo, embryo I+II was 2.46 ± 2.11, embryo III was 3.16 ± 2:11, embryo IV was 1.74 ± 2.18, the number of transferred embryo was 2.22 ± 0.61. A greater number (SD) of embryos I + II was observed in young patients (Table 1, Fig .1, P=0,027).

**Table 1:** Number of embryos (SD)/patients by age


Age	Embryos I+II/ patients

21-24	4.20
25-29	2.83
30-34	2.75
35-39	1.86
40-44	1.38
Total	4.2


We studied Pearson’s correlation coefficient for the markers of ovarian reserve. Evaluation of 120 patients showed a significant relationships between age and FSH (r=0.24, P=0.01), age and AMH (r=-0.22, P=0.02), age and collected oocytes (r=-0.23, P=0.03), age and metaphase II oocytes (r=-0.23, P=0.04), age and embryo I+II (r=-0.22, P=0.03), age and the number of transferred embryo (r=0.26, P=0.01, Tables2, 3, Fig .1). We also determined significant relationships between AFC and AMH (r=0.29, P=0.01), AFC and the number of transferred embryo (r=-0.18, P=0.03), AFC and total dose of the drugs (r=-0.23, P=0.03). Significant relationship was also observed between FSH and total dose of drugs (r=0.19, P=0.02) (Table 3).

**Table 2:** Spearman’s correlation of variables


Variables	Embryos* I	Embryos II	Embryos I+II	Embryos III	Embryos IV

Age	-0.11	-0.21**	-0.22**	-0.12	0.09
FSH	0.00	0.07	0.07	-0.03	0.03
AFC	0.01	0.05	0.05	0.01	0.14
AMH	0.12	0.08	0.11	0.04	0.10


*; Graded by Veeck’s criteria, **; Significant<5%, P<0.05, FSH; Follicle-stimulating hormone, AFC; Antral follicle count, and AMH; Anti-Mullerian hormone.

**Table 3:** Spearman’s correlation of variables


Variables	FSH	AMH	Number of collected oocytes	Number of metaphase II oocytes	Number of embryos	Number of em- bryo transferred	Total dose of drugs’ s stimulation

Age	0.24***	-0.22**	-0.23**	-0.23**	-0.13	0.26***	0.04
FSH		0.04	-0.05	0.00	0.02	0.10	0.19**
AFC		0.29***	0.16	0.09	0.11	-0.18**	-0.23**
AMH			0.38***	0.35***	0.19**	-0.13	-0.25***


**; Significant<5%, P<0.05, ***; Significant<1%, P<0.05, FSH; Follicle-stimulating hormone, AFC; Antral follicle count, and AMH; Anti-Mullerian hormone.

Other significant relationships were between AMH and the number of collected oocytes (r=0.38, P=0.01), AMH and the number of metaphase II oocytes (r=0.35, P=0.01), AMH and the number of embryo (r=0.19, P=0.04), AMH and total dose of the drugs (r=-0.25, P=0.01, Table 3).

## Discussion

In this investigation, we have validated the relationship between commonly used ovarian reserve clinical measures and outcome measures. 

Our observation, indicating that basal AMH, FSH and AFC are not related to embryo quality could contribute to an explanation for the low correlation with pregnancy probability, because embryo quality is crucial for clinical success (25). 

Although ovarian reserve markers have been shown to have some predictive power in the ART, there is consensus that they provide only general approximations of stimulation quantity (e.g. the number of oocytes retrieved in ART treatment cycles). The major limitations of these tests include their poor sensitivity and, in most cases, dependency on cycle stage. Furthermore, once a woman test is abnormal, her poor prognosis in ART is already established. 

Currently, there is no reliable test of ovarian reserve for an individual woman that could accurately predict her remaining reproductive life span. Integration of more than one marker improves the results, and repetition of some markers might be needed. 

An interesting point of this study is that average age of the group is 33 years, with 62.5% under 35 years, implicating that perhaps the oocyte quality can be impaired even before the age of 35.

Age was found to be predictive for the number of collected oocytes, number of metaphase II oocytes and embryo quality. This correlation of age and embryo quality could possibly be happened since oocyte is the major determinant of embryo developmental competence in women. It delivers half of the chromosomal complement to the embryo, but the maternal and paternal genomes are neither symmetrical nor equal in their contributions to embryo fate. Unlike the paternal, the maternal genome carries a heavy footprint of parental aging. This marker of ovarian reserve is the single best predictor of reproductive outcome in women, and oocyte is the locus of reproductive aging in women. The incidence of both whole chromosomal nondisjunction and precocious chromatid separation were correlated to maternal aging. Disturbance in sister chromatid cohesion might be a causal mechanism predisposing to premature chromatid separation and subsequently to nondisjunction in female meiosis. In addition, the asymmetry of female meiosis division could favor a nonrandom meiotic segregation of chromosomes and chromatids. 

An overall age-related change in the expression of certain genes and proteins, involved in mito- chondrial function, was observed in many stud- ies. Mitochondria play a primary role in cellular energetic metabolism, homeostasis, and cell death, while it is directly involved in oogenesis and foliculogenesis. Their functional status influences the quality of oocytes and sperm. It also contributes to the success of fertilization and embryonic development. Oocytes rely on ATP produced by the mitochondria via oxidative phosphorylation to generate energy. In the aging, there is increased mitochondrial DNA damage, a decrease in oxidative phosphorylation and ATP production for oocyte. Furthermore, mitochondrial mutations in follicular cells, surrounding the oocyte, have been correlated with maternal age, suggesting that oxidative phosphorylation in the follicle is compromised (26). 

Embryo quality may be affected by oxidative stress (27), but even morphologically normal embryos could show an abnormal number of chromosomes and low pregnancy rates (28). But perhaps the major factor in the etiology of age-related female infertility is decline in the oocyte quality associated with factors including, but not limited to, chromosomal aneuploidy and mitochondrial dysfunction (29, 30). However, the underlying mechanisms still remain poorly understood. 

Some limitations of the current investigation should be noted. First, relatively small sample size in this investigation may have limited our ability to demonstrate the additional value of these markers, related to embryo quality. Second, there are many published embryo scoring systems (31-36). Despite the systematic approach of such scoring methods to compare and contrast embryos, embryo morphology and assigning of a grade is, by default and design, a subjective process subject to interobserver and intraobserver variability, although all embryos were evaluated by the same embryologist.

We agree that the force of these clinical results described in a transparent manner are in the nonintentionality to fit a trended question. Instead of this, we took the results in order to analyse the presented data and make some recommendations based on it. The need for more simplified clinical treatments, cost reduction studies and dose of ovarian stimulation in countries without social coverage becomes imminent. Recent studies have aimed their proposal to focus on only markers of embryo quality (including age, AMH) and reduce the use of quantitative response markers (like FSH).

More studies have to be done to improve the accuracy and interpretation of the current ovarian reserve markers to state clear cut-off levels for each marker and find another markers which could more correlate with the number of ova retrieved, embryo quality and clinical pregnancy rate. Determining the etiology of maternal aging on oocyte competence could lead to improve patient care and fertility outcome. 

## Conclusion

We have demonstrated that commonly used clinical markers of ovarian reserve reflect true ovarian reserve and outcomes measures of ART, while age is the best predictor of embryo quality.
